# Is a purpose of REM sleep atonia to help regenerate intervertebral disc volumetric loss?

**DOI:** 10.1186/1740-3391-7-1

**Published:** 2009-01-05

**Authors:** Jerome CJ Fryer

**Affiliations:** 1 Private Practice, Nanaimo, British Columbia, V9S 3Y3, Canada

## Abstract

The nature of atonia in sleep continues to be enigmatic. This article discusses a new hypothesis for complete core muscle relaxation in REM sleep, suggesting a bottom-up recuperative perspective. That is, does the atonia in REM sleep provide a utility to help restore the mechanobiology and respective diurnal intervertebral disc hydraulic loss? By combining the effects of gravity with current compressive concepts in spinal stability, this article looks at vertebral approximation as a deleterious experience with an intrinsic biological need to keep vertebrae separated. Methods using polysomnography and recumbent MRI are discussed.

## Background

The goal of this article is to stimulate spine research in sleep. Specifically, I ask whether REM atonia plays a mechanical function in assisting recuperative imbibition to diurnally influenced cartilaginous structures in mammalian species. Since the discovery of REM sleep, researchers have been looking to the midbrain and surrounding parenchyma in search for answers with much progress in the neuro-mechanisms around the reticular formation. But do we know definitively the mechanical effects of REM's atonia on all diurnally influenced mammalian tissues? To the best of the author's knowledge, this relationship has not been thoroughly investigated and requires a closer look.

To appreciate the historical pursuit of REM sleep atonia's regulatory mechanisms, Michael Jouvet's study in 1962 [1] warrants honorable mention. Jouvet investigated subcortical activities in sleeping decerebrate cats. He measured EMG activity of neck muscles and found that muscle tone disappeared 4–5 times (for a period of about 6 min) over a 6 h course of sleep – even without the cortex. He also found that, during atonia, high voltage spiky waves appeared in the pontine EEG recording electrodes and waking EEG in the cortex. This apparent paradox (atonia and waking-like EEG activity) led him to coin the term "paradoxical sleep" and the research suggested the structures responsible for REM's characteristic identification of atonia were located caudal to the transection at the midbrain [2].

The function of REM sleep continues to be enigmatic [3], with atonia well documented in humans and animals. Some have explained this as the loss of core muscle tone [4] and by others, as the total paralysis of the anti-gravity muscles of the body [5]. But, to date, the best functional hypothesis for this complete pseudo-paralysis is believed to be for the purpose of not acting out our dreams. It is understood that this idea has evolved from the disturbing effects of REM sleep behavior disorders. Here, an alternative viewpoint and new hypothesis will be proposed.

**Figure 1:**
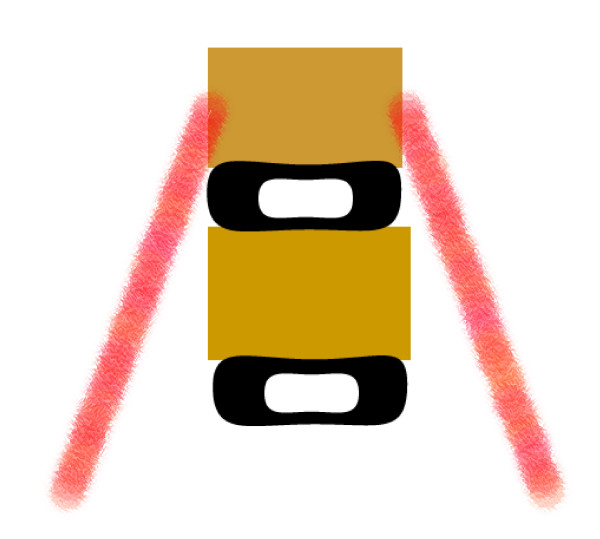
**Example of compressive muscle stabilization of the intervertebral disc**.

If the muscles of REM sleep atonia are identified as "antigravity muscles", then it would seem reasonable to understand clearly the gravitational influences on all adjacently associated mammalian structural tissues related to these muscles across day and night. The spine plays a foundational role in mammalian motility with associated spinal muscles attached to the vertebrae, by way of origins and insertions, and crossing intervertebral discs (IVDs) (see Figure 1, for example). Research on human IVDs and corresponding biomechanics has revealed a definitive nycthemeral variation of human stature [6]. It has been found that we lose height over the course of one day by as much as 26 mm, which is very likely due to changes in the IVDs. On average, 19.3 mm of height is lost with volumetric changes of 1300 mm^3^ to the lumbar discs [7]. Others have found in vivo daily variations of 16.2% in the lumbar IVDs [8] and 10.6% height gain over an 8 hr recumbent rest period with MRI [9]. In the absence of gravitational influences, some space flight studies have shown alterations in REM, suggesting a possible gravitational influence on sleep [10].

In order to consider this new functional hypothesis for REM's atonia, it is imperative that the reader understands current concepts of spinal stability. Simplistically, muscles can either contract or relax. Under the act of spinal stabilization, muscles contract to provide a 360 degree buttressing force to prevent the IVDs from buckling (see Figure 1). McGill [11] explains how the core musculature acts like guy wires of tension to create a stable platform. This concerted effort from the musculature around each spinal motion segment and across heavily water concentrated cartilaginous tissue causes a net effect of IVD compression. And, when this myotogenous spinal stability function is combined with the nycthemeral variation in the influence of gravity, the consequential pressures are thus significant on IVDs – showing their deformation with daily variations. Therefore, there are two combined forces working against the task of maintaining hydraulic vertebral spacing: 1) stabilizing musculature and 2) gravity. In absolute terms, an anti-gravity muscle is one that must create compression with the goal of minimizing approximation to osteological structures and associated articular cartilage. In other words, it is physically impossible to have a muscle that contracts against the forces of gravity. Using the term "anti-gravity muscles" may have been misleading terminology. The reader is directed to McGill's work for a full understanding of spinal biomechanics, but the highlights, for the purposes of this paper, have been mentioned.

It is proposed here that one of the possible reasons humans and most other mammals experience atonia during REM (and possibly during NREM) is to relax the compressive stabilizing musculature around the IVD(s) to allow effective imbibition of the cartilage in a pulse-like mechano-hydraulic fashion through sleep. This would simply aid to recuperate the net diurnal IVD height and related water loss experienced as a result of the loading effects on the preceding day. Relaxation of the muscles is proposed to result in net nutrient influx to these large avascular structures in mammalian species. Recent research has shown the importance of dynamic loads vs. static loads in oxygen delivery to IVDs [12] with the idea that this on/off pulse of REM atonia delivers varying loads and resultant mechanobiological influences to chondrocytes (and their microenvironments) across sleep.

## Some of what we know and what we don't know about REM and related topics

Approximately twenty-five percent of sleep is REM in the young adult [13] but interestingly, this varies with age [14]. We know that infants have much more REM compared to adults, as human infants typically enter REM sleep directly after the initial onset of sleep and spend approximately 50% of their total sleep time in REM [15]. And we also know that as we age, cartilaginous structures desiccate [16]. During development, infants have much more cartilaginous tissue compared to adults while they undergo the metabolic demanding task of endochondral ossification. Chondrocytes constitute the predominant cell of cartilage which is an interface tissue that is avascular, aneural and alymphatic [17]. These cells lay in an environment that is influenced by mechanical forces [18] whereby cellular perception of mechanical stress within cartilaginous tissues is an important modulator of chondrocyte function [19]. Recent understandings of growth plate proliferation have revealed that distraction is facilitatory while compression is inhibitory [20]. Therefore, could the relaxation of the mechanical tension across growth plates explain to provide a function of assisting growth and perhaps explain why infants have much more REM? Endochondral ossification related to growth is complete in humans around the ages of 18–24 yr which could possibly account for the difference in REM across the ages. The demand for micronutrients to these cellular processes in the adult may be less because there is less demand for the process of osseous growth.

Interestingly, cetaceans are the only mammals in which REM is not observed [21]. This finding could lend support to this new hypothesis. That is, aquatic mammals are not under the same gravitational demands as are land mammals and do not require the same buttressing spinal mechanisms for stability. They may not require atonia to recuperate the disc height loss in the same way land vertebrates do because of their aquatic environment. With minimal axial gravitational compressive loads coupled with the horizontal and constantly moving nature of their life, the need for atonia during REM could not be required.

Quadruped mammals are known to experience atonic sleep. And some readers may argue that the horizontal nature of quadrupeds would not require similar atonia to unload the upright bipedal nature of a human's spinal biomechanics. Comparative differences in sleep architecture are not too obvious; except that the size of a mammal appears to be related to the quantity of sleep [21]. It is believed that quadrupeds are designed with similar buttressing mechanics around the spine when compared to upright bipeds. Holding the fore limbs and hind limbs together must require similar compressive forces for stability to inhibit significant spinal bowing and vertebral approximation. And it is believed that the forces within the spine are more similar than different when comparing bipedals and quadrupeds. Unfortunately, the nycthemeral variations in the height and length of quadrupeds have not been investigated thoroughly. Perhaps, the small variations are difficult to detect. Horses, for example, can sleep standing in "stay mechanisms." But some authors have speculated that horses do not experience REM in this position but require lying down to experience REM (S McDonnell, e-mail communication, October 1, 2007). This observation would lend support to this hypothesis with horses requiring to lie down to spinally recuperate. Although unknowns regarding spinal nycthemeral variations in quadrupeds remain, further investigations may help define the physical aspects of recuperation during sleep in these mammals. Could the finding of mammal size and quantity of sleep be related to the size of the vertebrae and related hydraulic recovery?

## Suggested methods

**Figure 2:**
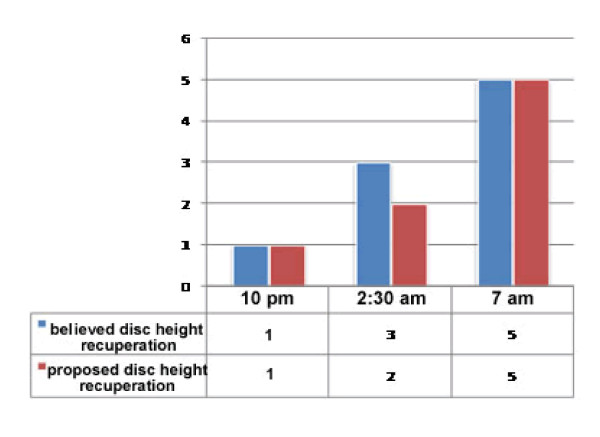
**Example of lumbar disc height, believed (blue) and proposed (red), across sleep**.

The testing methodology of whether spinal intervertebral disc restoration occurs significantly greater during REM sleep's atonia will likely require 3T MRI lumbar mapping and polysomnography. One method could involve obtaining three measures of lumbar IVD heights in sleep (10 pm, 2:30 am, and 7 am, for example) and correlating the changes to REM atonia. It is well known that there is a higher percentage of REM sleep in the latter half of the night and it is proposed that the atonia in REM will correlate to greater disc volume gains. Disc hydraulic recuperation has not been correlated to REM sleep. It is hypothesized that the atonia would contribute to a greater hydraulic recovery and in turn, provide insight into the diurnal hydraulic nature of spinal recuperation and its relationship to muscle tone in sleep. Aged matched controls would also be of interest, with an investigation across the full 24 hr cycle to be thorough. Figure 2 compares expected height changes under the proposed hypothesis with the height changes predicted by a linear model (as is suggested by previous work) over a bed-time cycle. Other methodological strategies could involve REM sleep deprivation and/or the use of medication to inhibit REM sleep with manipulation of independent variables. Importantly, age matched subjects who simply lie down and do not sleep (having MRI images) would prove as a useful control group. Additional tools like highly sensitive digital stadiometers could also help in revealing the answers to this new hypothesis. For other related works, see [22-26].

## Future directions

Because sleep is defined not as a single-state, but as a number of mixed states [2], it seems reasonable to approach the challenge of understanding *why we sleep *[27] through a careful dissection of all sleep's anatomy. Limiting our search to descending inhibitory neurological pathways with atonia may not allow us to step-back and look at all the requirements on Earth, including physical ones. Vertebral approximation is not a favourable situation in the spine [28] and, simplistically, it seems reasonable to think that there should be an innate biological mechanism to indicate when to allow IVDs to decompress and regain hydraulic structure and associated nutrients in preparation for the following day. The IVDs are daily oscillatory hydraulic structures that undergo compression throughout the waking day, but when they specifically recuperate their hydraulic loss in sleep has not been investigated thoroughly. Unfolding the full wake/sleep story on these biological tissues should help provide us with insights into concepts of physical recovery in sleep. Importantly, understanding the relationship of sleep to many medically related mobility disorders such as narcolepsy, cataplexy, fibromyalgia, Parkinsons, restless leg syndrome, and osteoarthritis, for example, will provide large rewards. The curious nature of atonia during REM may not be as complex as once thought. Simply, this mechanobiological investigation would look at whether or not REM atonia had an influence on water flow (with its solutes) into cartilage in sleep.

## Competing interests

The author declares that they have no competing interests.

## Authors' contributions

JF contributed to all of the article's content.
